# A Modular Millifluidic Platform for the Synthesis of Iron Oxide Nanoparticles with Control over Dissolved Gas and Flow Configuration

**DOI:** 10.3390/ma13041019

**Published:** 2020-02-25

**Authors:** Luca Panariello, Gaowei Wu, Maximilian O. Besenhard, Katerina Loizou, Liudmyla Storozhuk, Nguyen Thi Kim Thanh, Asterios Gavriilidis

**Affiliations:** 1 Department of Chemical Engineering, University College London, Torrington Place, London WC1E 7JE, UK; luca.panariello.15@ucl.ac.uk (L.P.); gaowei.wu@ucl.ac.uk (G.W.); m.besenhard@ucl.ac.uk (M.O.B.); katerina.loizou@gmail.com (K.L.); 2 Biophysics Group, Department of Physics and Astronomy, University College London, Gower Street, London WC1E 6BT, UK; l.storozhuk@ucl.ac.uk (L.S.); ntk.thanh@ucl.ac.uk (N.T.K.T.); 3 UCL Healthcare Biomagnetic and Nanomaterials Laboratories, 21 Albemarle Street, London W1S 4BS, UK

**Keywords:** nanomaterials, flow synthesis, gas–liquid reaction, carbon monoxide, hydrogen, membrane, tube-in-tube contactor

## Abstract

Gas–liquid reactions are poorly explored in the context of nanomaterials synthesis, despite evidence of significant effects of dissolved gas on nanoparticle properties. This applies to the aqueous synthesis of iron oxide nanoparticles, where gaseous reactants can influence reaction rate, particle size and crystal structure. Conventional batch reactors offer poor control of gas–liquid mass transfer due to lack of control on the gas–liquid interface and are often unsafe when used at high pressure. This work describes the design of a modular flow platform for the water-based synthesis of iron oxide nanoparticles through the oxidative hydrolysis of Fe^2+^ salts, targeting magnetic hyperthermia applications. Four different reactor systems were designed through the assembly of two modular units, allowing control over the type of gas dissolved in the solution, as well as the flow pattern within the reactor (single-phase and liquid–liquid two-phase flow). The two modular units consisted of a coiled millireactor and a tube-in-tube gas–liquid contactor. The straightforward pressurization of the system allows control over the concentration of gas dissolved in the reactive solution and the ability to operate the reactor at a temperature above the solvent boiling point. The variables controlled in the flow system (temperature, flow pattern and dissolved gaseous reactants) allowed full conversion of the iron precursor to magnetite/maghemite nanocrystals in just 3 min, as compared to several hours normally employed in batch. The single-phase configuration of the flow platform allowed the synthesis of particles with sizes between 26.5 nm (in the presence of carbon monoxide) and 34 nm. On the other hand, the liquid–liquid two-phase flow reactor showed possible evidence of interfacial absorption, leading to particles with different morphology compared to their batch counterpart. When exposed to an alternating magnetic field, the particles produced by the four flow systems showed ILP (intrinsic loss parameter) values between 1.2 and 2.7 nHm^2^/kg. Scale up by a factor of 5 of one of the configurations was also demonstrated. The scaled-up system led to the synthesis of nanoparticles of equivalent quality to those produced with the small-scale reactor system. The equivalence between the two systems is supported by a simple analysis of the transport phenomena in the small and large-scale setups.

## 1. Introduction

Magnetic nanoparticles represent a class of nanomaterials of particular interest in the biomedical field [1,2,3], with potential applications in thermal tissue ablation [4,5], immunoassays [6,7], magnetic resonance imaging [8,9,10,11], magnetic particle imaging [12], drug delivery [13,14,15,16], pathogen diagnostic assays [17] and tissue repair [18]. For these biomedical applications, iron oxide nanoparticles (IONPs) still represent the most promising magnetic nanomaterials due to their biocompatibility [1,4].

For the most common aqueous synthesis of IONPs, the so called co-precipitation method, the pH of a solution of ferrous and ferric ions is increased typically via the addition of a base, causing the precipitation of the nanoparticles [19,20]. This can happen either slowly by continuous addition over minutes or hours, or fast, i.e., via a one-time injection resulting in an abrupt pH increase. These commonly used fast co-precipitation syntheses result in the immediate formation of iron-containing particles after mixing the precursor and base solution, and depending on the synthesis conditions, these intermediate precipitates evolve towards the magnetite/maghemite phase [19,20,21]. The rapidity of such co-precipitation syntheses can limit the reproducibility [22] when using conventional batch reactors, where fast mixing is hard to achieve and precipitation kinetics might vary due to spatial pH variations. Micro- and millifluidic reactors have been proposed as an alternative to perform these syntheses, because fast and reproducible mixing conditions can be easily achieved due to smaller reactor dimensions. Nonetheless, the implementation of precipitation reactions in flow can be challenging, due to fouling or particle deposition on the reactor wall, causing material loss and eventually channel clogging. Abou-Hassan et al. [23,24] and Norfolk et al. [25] reported the use of a coaxial flow reactor, where the reaction takes place at the interface between two coaxial streams (i.e., the central and outer stream), for the co-precipitation of IONPs. Kumar et al. [26] reported the synthesis of 3.6 nm IONPs via a co-precipitation reaction using a droplet millifluidic reactor, where two aqueous streams containing iron precursor and ammonia were injected using two glass capillaries inside a polytetrafluoroethylene (PTFE) tube where an organic phase was flowing. The organic stream served as the continuous phase to prevent the aqueous solution from touching the wall. 

Among the biomedical applications involving the use of magnetic nanoparticles, magnetic hyperthermia stands out as complementary to conventional cancer therapy. Magnetic nanoparticles can heat up when exposed to an alternate magnetic field, allowing localized heat generation where particles accumulate, eventually leading to thermal ablation of the tumour. Focusing on magnetic heating of IONPs, high heating efficiency requires particles of size above 20 nm [27]. This is hardly achieved using conventional co-precipitation reactions [28]. A variant of the classic precipitation of ferrous and ferric salts was proposed by Sugimoto and Matijevic [29], and later modified by Vergés et al. [30], starting from only ${\mathrm{Fe}}^{2+}$ ions and adding a base in the presence of a mild oxidant such as ${\mathrm{KNO}}_{3}$. This synthesis (called oxidative hydrolysis of ${\mathrm{Fe}}^{2+}$ salts) yields particles with size that can be adjusted between 40 and 200 nm. The oxidative hydrolysis of ${\mathrm{Fe}}^{2+}$ is influenced by the reaction atmosphere composition, that can alter the redistribution between iron precursors ${\mathrm{Fe}}^{2+}$ and ${\mathrm{Fe}}^{3+}$, as well as directing the final product towards different phases. It is common practice to carry this reaction under air or inert atmosphere. However, recently, the use of reducing or oxidizing gas during the reaction showed the possibility of producing different products [31]. 

Gas–liquid reactions can be intensified with the use of flow reactors. This paradigm is widely accepted in the organic synthesis community, as gas–liquid segmented flow reactors are commonly employed to intensify small-molecule syntheses [32,33,34]. An alternative to gas–liquid segmented flow reactor is offered by membrane reactors, which allow the controlled and safe addition of gas to liquid via the use of gas-permeable membranes [35]. These devices are easier to scale up than gas–liquid segmented flow reactors, as the latter are limited by the channel size required to achieve stable segmented flow. Gas–liquid segmented flow reactors have been used in nanomaterial syntheses mainly to minimize the residence time distribution (RTD) width [36] or to improve the reactor mixing [37]. Only few works report the use of gas as both segmenting and reactive phase for the production of nanomaterials. Larrea et al. [31] demonstrated that different iron oxide phases and particle sizes can be achieved using CO, H_2_, $N_{2}$ or $O_{2}$ as segmenting gas in a gas-segmented flow reactor, while keeping the other synthesis parameters unchanged. This is likely due to the interaction of these gases with the reaction pathways, where a number of redox reactions involving the initially formed iron hydroxides (goethite and feroxyhyte) eventually lead to the magnetite/maghemite phase [38]. The same group recently exploited a similar experimental setup to synthesize iron-based nanomaterials decorated with catalytic nanoparticles [39]. Sebastian et al. [40] employed a gas–liquid segmented flow reactor to synthesize a library of different Pd and Pt nanomaterials by changing the type of gas employed to segment the reactive stream. The use of membrane gas–liquid contactors holds great promise to carry out gas–liquid synthesis in flow, as they allow for control over gas–liquid mass transfer and safe operation when using toxic gases (e.g., CO). However, only two works, to the best of our knowledge, have used this type of reactor for the synthesis of nanomaterials. Khan’s group used a liquid–liquid segmented flow reactor to synthesize gold nanoshells on silica using CO as reducing agent. The gaseous reactant was introduced in the reaction solution with a polydimethylsiloxane membrane [41]. Huang et al. [42] reported the synthesis of ${\mathrm{Au}}_{25}$ nanoclusters using a Teflon AF-2400 membrane tube-in-tube gas-liquid contactor, where a heptane stream was saturated with CO. The organic stream (the continuous phase) was used to segment the reactive aqueous stream, protecting the walls from potential deposition of particles and providing the reducing agent for the formation of the particles. 

In this work, we studied different flow configurations based on the assembly of a tube-in-tube gas–liquid contactor and a millifluidic reaction unit for the production of IONPs via oxidative hydrolysis of a ${\mathrm{Fe}}^{2+}$ salt, with the aim of producing pure magnetite/maghemite nanoparticles with size above 20 nm and value of saturation magnetization close to that of the bulk material for magnetic hyperthermia application. The use of a millifluidic platform (instead of a microfluidic one) allows us to increase the production rate while retaining the advantages provided by microreactors (i.e., high heat and mass transfer rates). The influence of flow configuration and gas dissolution were investigated. We also show the successful scale up of one of the reactors by a factor of 5 by increasing the reactor radius. The particles were then tested as potential magnetic heaters, exhibiting performance in line with literature.

## 2. Materials and Methods 

### 2.1. Materials

${\mathrm{FeSO}}_{4}\cdot 7H_{2}O$ (ACS reagent ≥99%) poly(acrylic acid) (average $M_{w}$ 1800), tetraethyl- ammonium hydroxide (TEAH, 35%_wt_ in $H_{2}O$) and heptane (HPLC grade ≥99%) were purchased from Sigma Aldrich (UK). $H_{2}{\mathrm{SO}}_{4}$ (0.05 M) and ${\mathrm{KNO}}_{3}$ (reagent grade ≥99%) were purchased from Honeywell Fluka (UK). Carbon monoxide was provided by BOC gases (UK), whereas hydrogen was produced using a hydrogen generator (PH600, Peak Scientific, Inchinnan, UK).

### 2.2. Particle Synthesis

**Figure 1:**
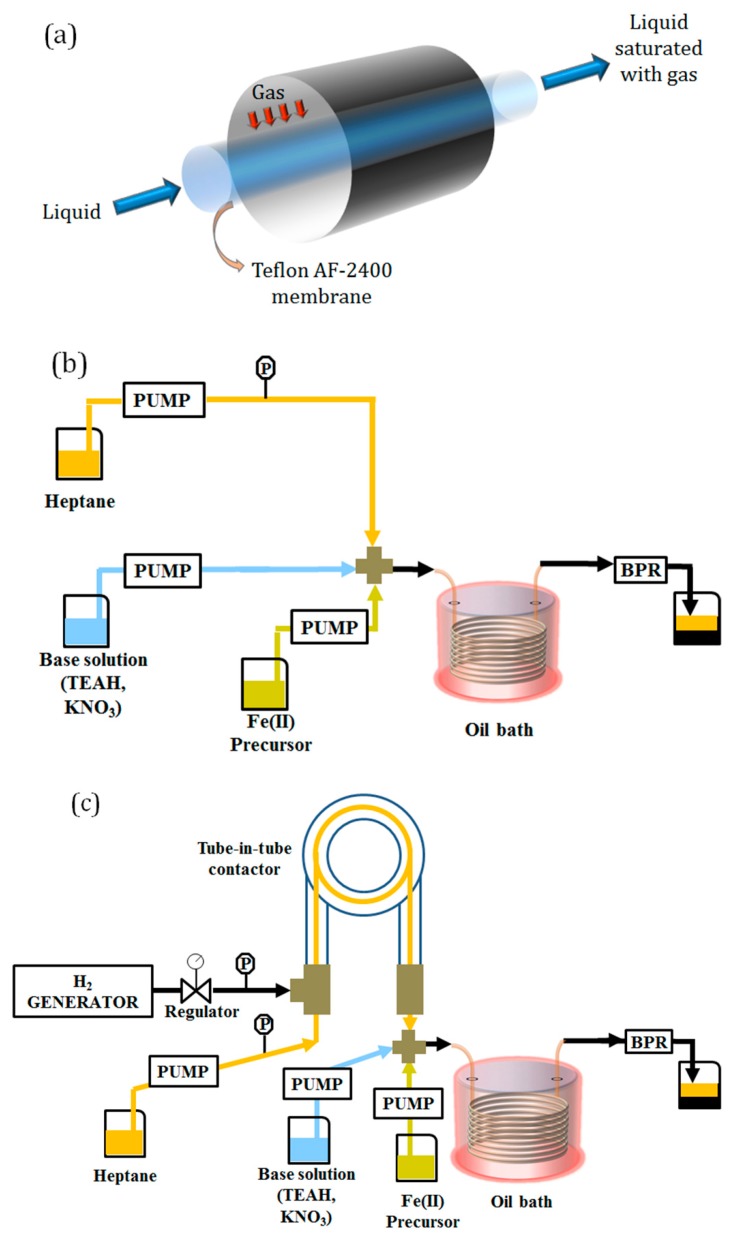

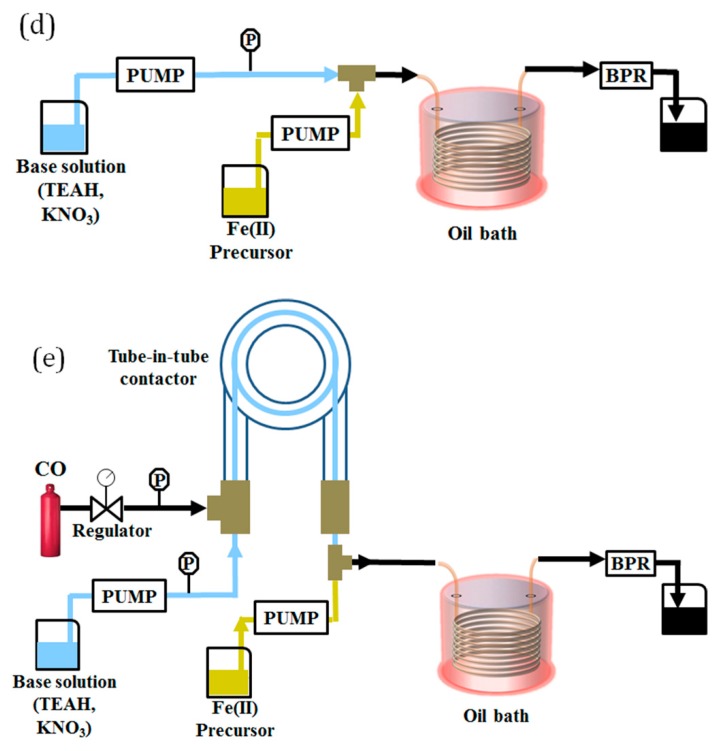
(**a**) Schematic describing the working principle of a tube-in-tube gas–liquid contactor. (**b**–**e**) Schematics of the setups employed for the flow synthesis. (**b**) Reactor System 1, segmented flow reactor with solutions preventively bubbled with $N_{2}$; (**c**) Reactor System 2, segmented flow reactor with $H_{2}$-saturated heptane; (**d**) Reactor System 3, single-phase reactor with solutions preventively bubbled with $N_{2}$; (**e**) Reactor System 4, single-phase reactor with CO-saturated base solution.

Four flow reactor systems were designed based on the assembly of a tube-in-tube gas–liquid contactor and a millifluidic reaction unit. The tube-in-tube gas–liquid contactor was similar to that used by Huang et al. [42], and was made of a 2 m long gas-permeable Teflon AF-2400 tube (0.8 mm inner diameter (ID), 1 mm outer diameter (OD), Biogeneral, San Diego, CA, USA) placed inside a polytetrafluoroethylene (PTFE) tube (2.4 mm ID, 3.2 mm OD, Thames Restek, High Wycombe, UK) and assembled together by a T-junction (hole size: 1.0 mm, Upchurch Scientific, Oak Harbor, WA, USA) and a union (hole size: 1.0 mm, Upchurch Scientific, Oak Harbor, WA, USA). The contactor was used to saturate a liquid stream fed in the Teflon AF-2400 tube with different gases (either CO or $H_{2}$) that were pressurised in the annulus between the inner and outer tube. A 3 bar pressure in the gas side was maintained by a gas pressure regulator (K type, Swagelok, Solon, OH, USA) and monitored by a pressure sensor (40PC150G, Honeywell, Charlotte, NC, USA) placed at the inlet of the annulus. A schematic of the gas–liquid contactor is shown in Figure 1a. The millifluidic reaction unit consisted of a PTFE coil of 6 mL (1 mm ID, ~7.65 m long, Thames Restek, High Wycombe, UK) maintained at 110 °C with the aid of a hotplate (RCT Basic, IKA, Staufen, Germany) which heated a stirred silicon oil bath. The temperature inside the bath was measured at different points with the aid of a thermometer to ensure temperature uniformity. 

In all the syntheses carried out, particles were obtained by mixing two aqueous streams—one containing the iron precursor and sulphuric acid and the other containing the base and the oxidant. The concentrations used were adapted from Larrea et al. [31] with minor modifications. After mixing, the concentration of the various components were: $\left[{\mathrm{Fe}}^{2+}\right]$ = 6.5 mM, $\left[H_{2}{\mathrm{SO}}_{4}\right]=1.69\;\mathrm{mM},\;\left[\mathrm{TEAH}\right]=81\;\mathrm{mM}$ and $\left[{\mathrm{KNO}}_{3}\right]=90\;\mathrm{mM}$. For all syntheses, all solutions were purged with nitrogen for at least 45 min before being transferred to the syringes employed for their pumping.

Four different reactor systems were used:

- Reactor System 1: this configuration comprised a liquid–liquid segmented flow reactor, where the organic phase (heptane) wets the reactor wall and segments the aqueous stream. A segmented flow pattern was achieved (size of slugs/droplets ~4 mm) by mixing the streams containing the iron precursor with the base solution and the organic stream in a polyether ether ketone (PEEK) cross junction (1 mm ID, Upchurch Scientific, Oak Harbor, WA, USA). The flow rate of the organic stream was set to 1 mL/min, while equal flow rates of 0.5 mL/min were used for the iron precursor and the base solutions. In this system, the gas–liquid contactor was not used, as the solutions were preventively bubbled with $N_{2}$ (inert gas). A schematic of the setup is shown in Figure 1b.
- Reactor System 2: this configuration comprised the tube-in-tube gas–liquid contactor upstream of the reaction coil. In the tube-in-tube contactor, an organic stream (heptane) was saturated with $H_{2}$. The heptane stream was used to segment the aqueous one before entering the reactor. The two water solutions (base and iron precursor) were mixed and segmented in a PEEK cross junction (1 mm ID, Upchurch Scientific, Oak Harbor, WA, USA). The flow rate of the organic stream was set to 1 mL/min, while equal flow rates of 0.5 mL/min were used for the iron precursor and the base solutions. A schematic of the setup is shown in Figure 1c.
- Reactor System 3: this configuration consisted of a single-phase reactor where the two reactant streams containing the iron precursor and the base were mixed in a PEEK T-junction (1 mm ID, Upchurch Scientific, Oak Harbor, WA, USA) before entering the reaction stage. The overall flow rate was set to 2 mL/min, and each aqueous stream was pumped at 1 mL/min. In this case, the gas–liquid contactor was not used, as the solutions were preventively bubbled with $N_{2}$ (inert gas). A schematic of the setup is shown in Figure 1d.
- Reactor System 4: this configuration consisted of a single-phase reactor. Here the base solution passed through the tube-in-tube contactor at a flow rate of 0.5 mL/min, where it was saturated with CO, and then mixed with the solution of iron precursor (1.5 mL/min). The two solutions were mixed in a PEEK T-junction (1 mm ID, Upchurch Scientific, Oak Harbor, WA, USA) before entering the reaction stage. A schematic of the setup is shown in Figure 1e.

The liquid pressure was maintained at 3.5 bar for all reaction systems with the aid of a back pressure regulator (K type, Swagelok, Solon, OH, USA) placed at the reactor outlet, and it was monitored with a pressure sensor (Little Things Factory GmbH, Elsoff, Germany) placed either upstream of the tube-in-tube contactor when used (i.e., Reactor Systems 2 and 4), or at the heptane stream before the cross junction in Reactor System 1, or at the base stream before the T-junction in Reactor System 3. 

The residence time τ in all millifluidic reactors was set equal to 3 min. For the two-phase flow Reactor Systems (Reactor Systems 1 and 2), we define the nominal residence time $\tau =V/Q_{tot}=V/\left(Q_{aq}+Q_{org}\right)$, where V is the volume of the reactor, $Q_{aq}$ is the total flow rate of the aqueous phase and $Q_{org}$ is the flow rate of the organic phase. For the single-phase flow Reactor Systems (Reactor Systems 3 and 4), $\tau =V/Q_{aq}$. Mixing time was estimated from Falk and Commenge [43], who reported the mixing time as a function of Reynolds number for a number of different micromixers. The mixing time was estimated to be *ca.* 8 s, and in all cases the residence time between the mixing junction and the millifluidic reactor was set equal to 10 s. The aqueous solutions were pumped by means of syringe pumps (Legato 210, KD Scientific, Holliston, MA, USA) using 50 mL glass syringes (Scientific Glass Engineering, Milton Keynes, UK). In the configurations where the two aqueous streams had the same flow rates (Reactor Systems 1, 2, 3), a single two-channel syringe pump was used, whereas when different aqueous flow rates were used (Reactor System 4), two different syringe pumps were employed. Heptane was pumped using a HPLC pump (Azura P 2.1S, Knauer, Berlin, Germany). After the synthesis, the particles were collected by magnetic separation, re-dispersed in an equal volume of a poly(acrylic acid) (0.72 mg/mL_H_2_O_) solution, which acted as a stabilizer and mechanically stirred for 30 min.

### 2.3. Reactor Scale Up

Reactor System 3 was scaled up by a factor of 5 by increasing the reaction coil volume. The small-scale 6 mL coil was changed with a 30 mL PTFE coil (2.4 mm ID, Thames Restek, High Wycombe, UK). The temperature and the residence time were kept the same as for the smaller scale reactor, hence the flow rate was increased by a factor of 5 (overall flow rate after mixing 10 mL/min). The solutions were pumped with the same syringe pump as in system 3 but using 100 mL glass syringes (Scientific Glass Engineering, Milton Keynes, UK). In order to avoid boiling of the solution, the system was pressurized to 1.5 bar with the aid of a back pressure regulator (K type, Swagelok, Solon, OH, USA).

### 2.4. Particle Characterization

Particle size and morphology were determined from transmission electron microscope (TEM) images, acquired using a JEOL 1200 EX TEM (Tokyo, Japan) with a 120 KV acceleration voltage. Particle crystal structure was determined via X-ray diffraction (XRD) using a PanAlytical X-ray diffractometer (X’Pert Pro, Malvern, UK) with Co Kα radiation (λ = 1.789 Å). Magnetic properties were determined via vibrating sample magnetometry (VSM, Model 6000, Physical Property Measurement System, Quantum Design, San Diego, CA, USA). Samples for XRD and VSM were prepared by isolating the particles from solution with a magnet and letting them dry in open air. Iron concentration was determined via microwave plasma atomic emission spectroscopy (4210 MP-AES, Agilent, Santa Clara, CA, USA). Before the analysis, the particles were separated from the supernatant solution with a magnet and digested with aqua regia (HCl 37%:HNO_3_ 70% 3:1 v:v, *Caution! Aqua Regia is a very corrosive oxidizing agent and should be handled with great care*), then diluted with water with a final volumetric ratio of 1:10 aqua regia: water. DLS studies were performed using a DelsaMax-Pro (Beckman Coulter, Brea, CA, USA) at 22 °C to determine the particles hydrodynamic diameter. For the measurements, the samples were diluted with deionized (DI) water until the measured sizes plateaued (typically at sample solution: DI water ratios < 1:4 v:v). Heating curves under alternating magnetic field (AMF heating) were measured using an alternating magnetic field applicator equipped with a fibre optic for temperature reading (G2 driver D5 Series, nB nanoScale Biomagnetics, Zaragoza, Spain). 

## 3. Results and Discussion

### 3.1. Two-Phase Flow Reactor Systems

In Reactor System 1, liquid–liquid segmented flow was utilized, where heptane was used to segment the aqueous stream, producing droplets, each acting as a “travelling” batch reactor. The reactor was operated at a temperature of 110 °C, hence above the boiling point of water. This is easily and safely achievable in flow due to the ability of using back-pressure regulators to control the pressure of the system, hence giving access to a window of operating parameters hardly accessible in batch. The use of temperatures above the solvent boiling point together with the high heating rates typical of flow reactors allow for significant improvements in the synthesis of small molecules [44], in particular significantly reducing reaction times.

**Figure 2:**
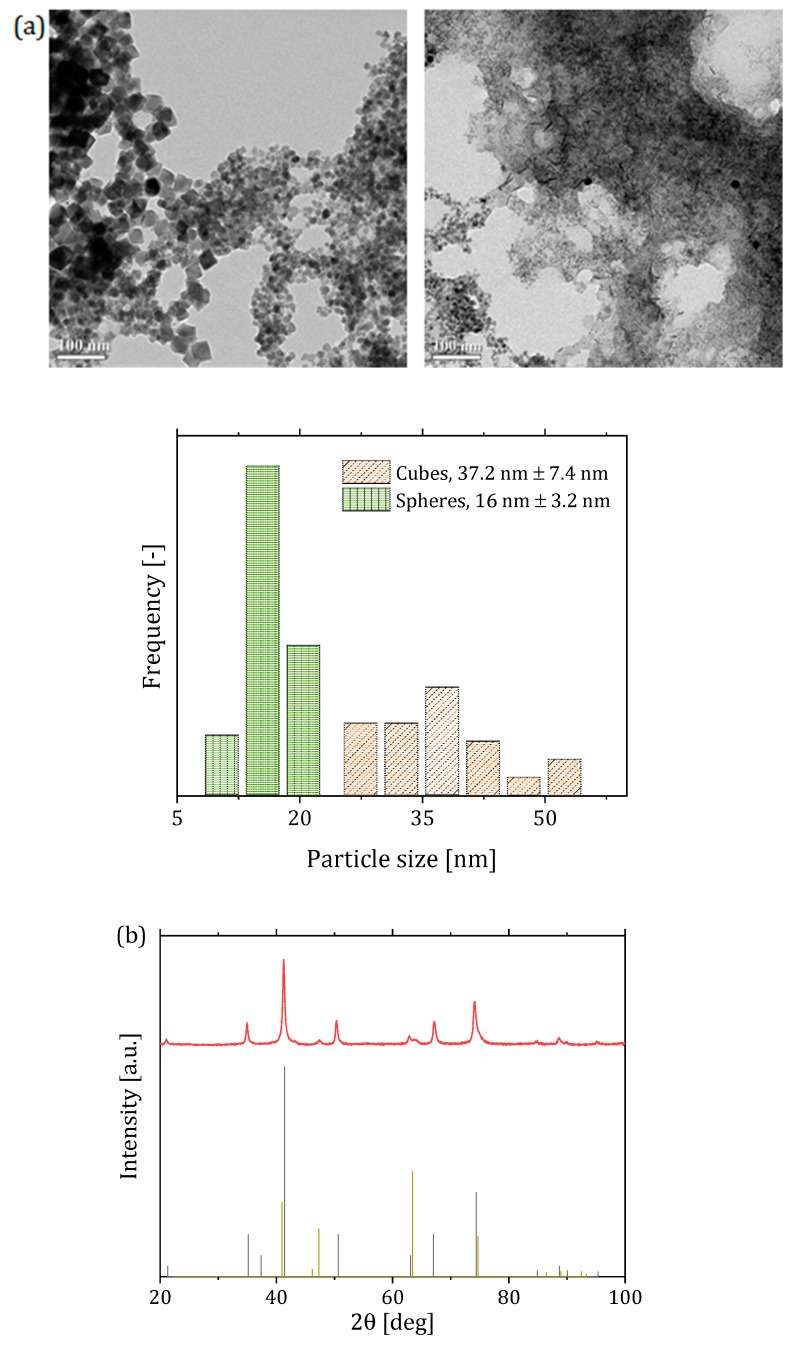
(**a**) Transmission electron microscope (TEM) micrographs of particles obtained from Reactor System 1, showing the presence of three different particle populations, with cuboidal, spherical and nanoflake morphology. Scale bar = 100 nm. The histogram reports the particle size distribution of spherical and cuboidal particles from Reactor System 1. Nanoflake particle size distribution was not determined due to strong aggregation between particles on the grid; (**b**) X-ray diffraction (XRD) pattern for the particles produced using Reactor System 1, showing the two identified phases: magnetite/maghemite (black vertical lines, pdf ref. 03-065-3107) and feroxyhyte (olive vertical lines, pdf ref. 01-077-0247).

The synthesis led to the production of polydisperse particles with the presence of three different populations, characterized by large particles with cuboidal morphology, smaller spherical particles and nanoflakes (Figure 2).

Even though most of the particles produced were crystalline magnetite/maghemite (see Figure 2 and Figure S1), we observed the presence of nanoflakes resembling those obtained in inert atmosphere at short reaction time by Larrea et al. [31]. These were reported to correspond to a mixture of goethite and feroxyhyte. Features of feroxyhyte were also observed in the XRD spectra of these particles (Figure S1). These impurities represent a small fraction of the product, as their contribution in the XRD pattern is very low. Larrea et al. showed that the goethite and feroxyhyte particles disappear during the synthesis, being incorporated in the magnetite particles and converted to the magnetite/maghemite phase. The authors observed the complete conversion of the iron precursor upon mixing, with the formation of a mixture of phases that then disappeared during the reaction. This is in agreement with our MP-AES results, where complete conversion of the iron precursor was observed (98%). A similar mechanism, though exhibiting different intermediate phases, was recently reported for the co-precipitation of iron chlorides salts with sodium bicarbonate as base [19]. 

**Scheme 1:**
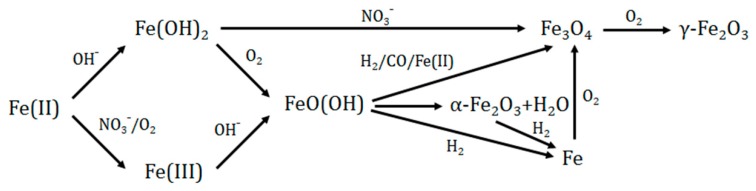
Reaction network leading to the formation of magnetite/maghemite based on the works of Correa et al. [38] and Larrea et al. [31]. Partially adapted from [38] with permission from Elsevier.

Based on the close resemblance between our results and those obtained by Larrea et al., we attempted to increase the reaction rate using H_2_, a reducing gas that has been shown to accelerate this synthesis [31]. Scheme 1 summarizes the reactions proposed for the synthesis of magnetite/maghemite nanoparticles starting from Fe(II), considering the contribution of gaseous reactants, such as $H_{2}$ and CO, based on the works from Correa et al. [38] and Larrea et al. [31]. The scheme shows how $H_{2}$ helps in reducing feroxyhyte to magnetite, as feroxyhyte can be directly reduced to magnetite or reduced to iron (which is then oxidized to magnetite).

**Figure 3:**
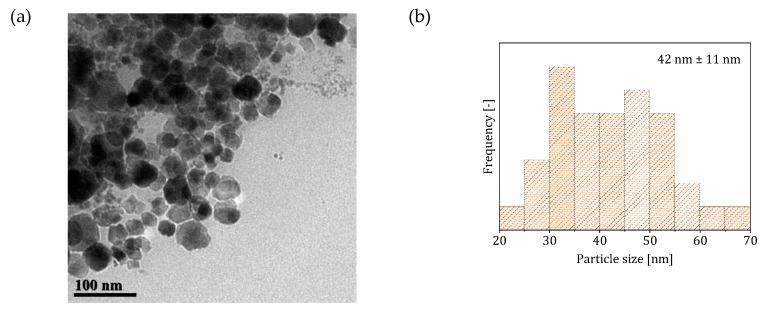
(**a**) TEM micrograph and (**b**) particle size distribution of particles obtained from Reactor System 2.

We introduced hydrogen by saturating the heptane stream in the tube-in-tube gas–liquid contactor. After the contactor, the organic stream segments the combined aqueous streams (Reactor System 2, Figure 1c), generating the segmented flow pattern. The hydrogen concentration profile in the tube-in-tube contactor was simulated similarly as in Yang and Jensen [35] (see Table S1, Figure S2). The results of the simulations show that, after approximately 25% of the contactor length, the heptane was saturated with H_2_, with a final concentration of 13.3 mM. The reactor allowed the synthesis of IONPs with full precursor conversion in only 3 min (99% conversion based on MP-AES), whereas typically this reaction is carried out in batch for as long as 24 h [30]. The XRD pattern of the produced particles did not show any sign of feroxyhyte (Figure S1) and no nanoflakes were observed in the TEM pictures (Figure 3), suggesting a higher purity for this sample towards magnetite/maghemite. This feature could be attributed to the effect of $H_{2}$ during the synthesis.

TEM micrographs revealed a different particle morphology than that obtained from Reactor System 1—spherical rather than cuboidal (Figure 3). The size distribution had an average size of 42 nm, and appeared rather wide (~26% relative standard deviation). Compared to Reactor System 1, the obtained particles appear more monodisperse, with a single population of particles, against the three distinct populations observed in Reactor System 1. The loss of the cuboidal shape, characteristic of this synthesis in batch under similar conditions (pH and reactant concentrations) [30,31,45], as well as the high polydispersity of the particles, may be due to interfacial adsorption affecting the synthesis. This phenomenon is the accumulation of solid particles at the interface between two liquids, driven by the minimization of the interfacial energy [46]. Zhang et al. [47] showed that, due to interfacial adsorption, asymmetric nanostructures can be generated, significantly different from those obtained in batch. The same authors also observed an increase in the particle polydispersity compared to batch, with the formation of two distinct particle populations, due to the generation of different concentration profiles within the droplet between the metal precursor and the particles. 

The particles produced using Reactor System 2 exhibited a hydrodynamic diameter of 247 nm. No sign of sedimentation was observed for ~2 h. One week after their synthesis, the particles could be redispersed after sedimentation by sonication, showing similar DLS results, whereas one month after their synthesis the particles could hardly be redispersed even with sonication, sedimenting after few minutes. 

Both Reactor Systems 1 and 2 allowed for the production of 0.36 mg/mL of iron oxides, with a production rate of 21.6 mg/h. The maximum collected volume during a single reactor run was ~70 mL, value limited only by the size of the syringes.

### 3.2. Single-Phase Flow Reactor Systems

**Figure 4:**
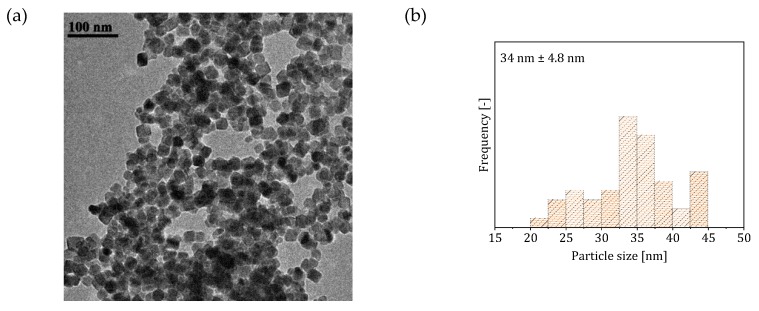
(**a**) TEM micrograph and (**b**) particle size distribution of particles obtained from Reactor System 3.

In light of the disadvantages encountered when using segmented systems, we decided to attempt the same synthesis in a single-phase reactor. Uson et al. [48] showed that, using sufficiently high flow rates, channel blockage can be avoided during the synthesis of iron oxide nanoparticles. Furthermore, at high pH, the zeta potential of PTFE is significantly negative [49], protecting the walls from particles adsorption during the synthesis (as the particles produced with this synthesis exhibit a negative surface charge [30]). Based on these observations, we carried out the synthesis in a single-phase reaction system (Reactor System 3) tuning the flow rate with sufficiently high shear to avoid particle deposition. Using an overall flow rate of 2 mL/min neither deposition nor channel blockage were observed during operation (maximum of 50 min due to limitations imposed by the volume of the syringes). The reactor allowed full precursor conversion in 3 min residence time (98% conversion based on MP-AES analysis). TEM images (Figure 4) show cuboidal particles with an average size of 34 nm and a narrow size distribution (relative standard deviation of ~15%). The uniform cuboidal structure and the narrow size distribution, compared with those obtained from Reactor Systems 1 and 2, further support the hypothesis of interfacial adsorption during the synthesis in two-phase flow systems. The XRD patterns of these particles exhibit only features of magnetite/maghemite (Figure S1). This, together with the uniform particle morphology observed via TEM (with no signs of nanoflakes), suggests good purity towards the magnetite/maghemite phase.

**Figure 5:**
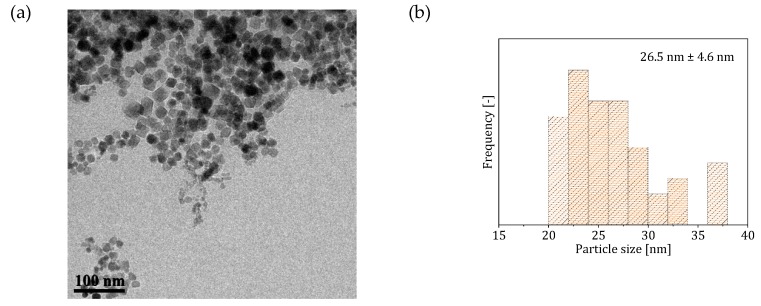
(**a**) TEM micrograph and (**b**) particle size distribution of particles obtained from Reactor System 4.

Carbon monoxide adsorption on the nanoparticle surface is reported to influence the particle structure and morphology for other nanomaterials, such as Pt and Pd [50]. Since the synthetic protocol used here does not involve a specific ligand, we envisioned the possibility to control the size of the produced IONPs by introducing CO in the single-phase reactor. The gaseous reactant saturated the base solution before mixing with the iron precursor solution using the tube-in-tube contactor. In order for the gas to saturate the base stream, its flow rate had to be reduced to at least 0.5 mL/min (compared to 1 mL/min used in Reactor System 2, see Figure S2). This is due to the lower driving force for gas transfer in Reactor System 4 as compared to Reactor System 2. Based on our calculations, when using a flow rate of 1 mL/min through the tube-in-tube contactor in Reactor System 4, the concentration of dissolved CO in the base stream reaches only half of the saturation value expected at the system temperature and pressure. In order to maintain the same overall flow rate in the heated coil (after reactant mixing), two different pumps were used, one eluting the base solution at a flow rate of 0.5 mL/min to the tube-in-tube contactor, and the other one eluting the iron precursor solution at a flow rate of 1.5 mL/min directly to the T-mixer prior to the reaction stage. The concentrations of the two solutions (base and iron precursor) were adjusted in order to maintain the same final concentration after mixing, as in the other reactor systems. No particle deposition at the reactor walls during operation was observed. Reactor System 4 allowed full precursor conversion in only 3 min residence time as the other reactor systems (97% conversion based on MP-AES analysis). TEM images (Figure 5) revealed smaller particles than those obtained using Reactor System 3 (average size of 26.5 nm against 34 nm from Reactor System 3), retaining a cuboidal morphology. No nanoflakes were observed on the TEM grid. XRD showed only features of magnetite/maghemite (Figure S1), suggesting these phases as the major constituent of the sample.

In addition to the adsorption on the nanoparticle surface, Larrea et al. [31] showed that CO can also alter the synthesis redox mechanism depending on the reaction temperature, with magnetite obtained only when carrying out the reaction at high enough temperature (100 °C). At this temperature, catalytic activity from FeOOH in CO oxidation is observed [51], supporting the behaviour of CO as reducing gas during the synthesis reported here. The reaction was carried out at 110 °C, avoiding the formation of phases different from magnetite/maghemite.

Particles produced using Reactor Systems 3 and 4 exhibited a hydrodynamic diameter of 220 nm and 198 nm respectively (based on DLS). The colloidal solutions produced from these reactors showed no visual sign of sedimentation for ~2 h, and could be redispersed after sedimentation through sonication, giving similar DLS signals. One month after their synthesis, the particles could still be redispersed by sonication.

Both Reactor Systems 3 and 4 allowed for the production of 0.36 mg/mL of iron oxides, with a production rate of 43.2 mg/h. The maximum collected volume during a single reactor run was ~70 mL, value limited only by the size of the syringes.

### 3.3. Magnetic and Heating Properties of the Nanoparticles

The magnetic properties of the IONPs produced using Reactor Systems 2, 3 and 4 were determined via VSM (Figure S3). All samples exhibited ferromagnetic behaviour (see SI 3) and values of saturation magnetization were ~80 emu/g_sample_, close to that of bulk magnetite (92 emu/g). Slightly higher values were measured for the particles obtained in Reactor System 2 (liquid–liquid segmented flow with H_2_ -saturated heptane). This might be due to the larger particle size obtained from Reactor System 2, and possibly to a higher fraction of magnetite than maghemite in the particles thanks to the excess of H_2_ used during the synthesis ($[H_{2}]/\left[{\mathrm{Fe}}^{2+}\right]∼2$). 

In light of their size and magnetic properties, the particles produced using Reactor Systems 2, 3 and 4 appeared as good candidates for magnetic hyperthermia. The heating performances of these particles were tested under exposure to an alternate magnetic field at two different conditions of field frequency f and strength H, namely f = 303 kHz and H = 24.6 kA/m and f = 759 kHz and H = 19.9 kA/m. The samples were placed inside an evacuated sample holder, and the temperature was recorded with the aid of a fibre optic placed inside the solution for 180 s (see Figure S4). For each heating experiment, both the specific absorption rate SAR (given by the heating rate of the sample normalized over the mass of particles present in solution) and the intrinsic loss parameter $\mathrm{ILP}=\mathrm{SAR}/fH^{2}$ were calculated using the corrected slope method [52]. The results obtained are summarized in Table 1.

The values of SAR obtained from Reactor Systems 3 and 4 are in line with those from the literature using particles produced through similar syntheses [45,53,54], with values ranging from ~200 W/g_Fe_ to ~400 W/g_Fe_ when increasing the frequency, corresponding to an ILP ~1.3 nHm^2^/kg. The values of SAR of the particles produced using Reactor System 2 were instead higher, ranging from $501\;W/g_{\mathrm{Fe}}$ to $765\;W/g_{\mathrm{Fe}}$ when increasing the frequency, with an ILP ~2.7 nHm^2^/kg. This value approaches those obtained in multicore particles [55] and Fe@Fe_x_O_y_ core-shell particles [56]. The higher ILP measured is unlikely to be related to the higher values of magnetization measured for these particles, as the saturation magnetization between the various particles varies by ~3%, and this variation appears too small to justify such an increase in the ILP. The higher ILP obtained from the particles produced from Reactor System 2 is more likely due to their size. The particles produced from this reactor exhibit a larger particle size, with a significant fraction of particles equal or above 40 nm compared to those obtained from Reactor System 3 and 4. Tong et al. [27] showed that iron oxide nanoparticles exhibit a sigmoidal dependency of the heating rate on the particle size, saturating at ~40 nm, where the maximum heating rate is achieved. The higher ILP could also be due to the change of the magnetic anisotropy of the IONPs produced from Reactor System 2 [57,58]. 

### 3.4. Scaled-up Reactor System

**Figure 6:**
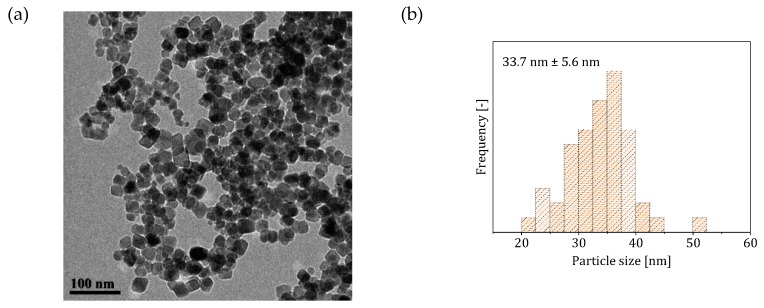
(**a**) TEM micrographs and (**b**) particle size distribution of particles obtained from the large-scale version of Reactor System 3.

For millifluidic reactors to become an appealing alternative to conventional batch reactors at actual manufacturing scale, the productivity of the reactors needs to be increased by increasing their volume while at the same time maintaining the advantages of millifluidic devices. Even though the particles produced from Reactor System 2 exhibited the highest ILP, because of their lower stability compared to the other samples, we decided to pursue the scale up of one of the single-phase reactor systems. As the particles produced from Reactor System 3 and 4 exhibited similar performance, we attempted the scale up of Reactor System 3, which required the simplest lab setup. The scale up was performed by increasing the tube internal diameter from 1 to 2.4 mm while decreasing the tube length from 7.6 m to 6.6 m, with an overall reactor volume increased by a factor of 5, keeping the average residence time constant. MP-AES results showed that the scaled-up reactor provided full iron precursor conversion as its smaller scale counterpart (large-scale system conversion equal to 98% based on MP-AES analysis). TEM micrographs (Figure 6) revealed that the particle size distribution and morphology was not affected by the change in reactor scale, with the large-scale reactor leading to particles of 33.7 nm (±5.6 nm), against the 34 nm (±4.8 nm) obtained from the small-scale reactor. DLS measurements also showed a similar hydrodynamic diameter, with the particles obtained from the large-scale system having a diameter of 235 nm against 220 nm of the particles produced from the small-scale system. The XRD patterns of the particles produced using the large-scale reactor appear equivalent to that of the particles produced using the small-scale system (Figure S5a), suggesting that the scale up did not affect the selectivity of the system towards magnetite/maghemite. The particles produced by the large-scale reactor system were also tested for AMF heating, showing similar values of SAR (Figure S5b).

To understand why the product of the scaled-up reactor remains almost unchanged compared to that of the smaller scale reactor, we investigated the transport phenomena in the reactors. Let us define the reactor length as L, its diameter as $d_{t}$, the average fluid velocity as u, the molecular diffusivity of the reactants (i.e., 2 nm iron oxide nuclei [59]) as $D_{m}$, the axial dispersion coefficient as $D_{ax}$, the liquid density and viscosity as ρ and μ respectively. The first quantity one must analyse is the Reynolds number ($Re=\rho ud_{t}/\mu$). After calculating this quantity, we can conclude that both reactors (small and large scale) exhibit laminar flow; Re increased with the increase of the reactor scale from 42 to 88. For a detailed description of the reactor behaviour, it is necessary to know the properties of the reactants and those of the particles during the synthesis [60], as their diffusivity is size-dependent. We can assume that all the precursor reacts upon mixing with the base [31] causing the precipitation of a mixture of phases, and then the particles grow via an aggregative process, evolving towards the final stable magnetite/maghemite phase [19]. The key aspect to explain the behaviour of the reactor is then the temporal evolution of the particles, unknown in this specific case. However, one can analyse two “extreme” cases, considering or neglecting particle Brownian motion. Colloidal nanoparticles in general exhibit Brownian motion. However, multicore particles can form during co-precipitation reactions [22], hence their diffusivity is expected to be much lower than that of small molecules. In the first case (non-negligible diffusivity), assuming a diffusivity of $D_{m}∼3\times 10^{-10}\;m^{2}/s$ for the reactants (i.e., 2 nm iron oxide nuclei, obtained from Stokes-Einstein equation), we calculated the Bodenstein number ($Bo=ud_{t}/D_{m}$, ranging from 1.5 × 10^5^ and 3 × 10^5^ for the small- and large-scale system respectively). These values together with the reactor aspect ratio ($L/d_{t}∼3000$ and ~8000 respectively for the large and small-scale system) indicate that both small- and large-scale reactors behave in good approximation accordingly to the Taylor–Aris model [60,61]. Since both reactors are characterised by very large values of Bo, one can approximate $D_{ax}=u^{2}d_{t}^{2}/192D_{m}$ [61], resulting to values of the axial dispersion number D/uL from ~0.05 to ~0.2 as the reactor scale increases. The increase does not appear dramatic, but could explain the slight increase in the relative standard deviation of the particles produced with the larger scale reactor, as a higher axial dispersion number translates in a broader RTD, which in turn causes a broader particle size distribution [60]. However, this would apply only for particles with a size of 2 nm. This is not the case, and since the diffusivity of the particles scales inversely with their hydrodynamic diameter, the actual value of diffusivity could be much higher than that adopted, up to a point where no Brownian motion takes place and the transport of these aggregates is only due to the drag exerted by the fluid flow. This situation represents the second “extreme” case. The combined increase in tube diameter and flow rate result to an average fluid velocity approximately constant (changing from 40 to 37 mm/s when scaling up the reactor), whereas the value of Re remains of the same order of magnitude. The drag force exerted from the fluid on the particles is proportional to $u^{2}C_{D}$, where $C_{D}$ is the drag coefficient. The latter depends on the Re number, and in the range analysed here has values from 1.6 to 1.3 when going from the small- to the large-scale reactor [62]. Considering that the residence time remained constant when scaling-up the system, and that the length of the reactor reduced from 7.6 to 6.6 m, the ratio between the drag force exerted on the particles in the small- and large-scale reactor is ~1.6, hence the drag does not differ significantly between the two systems. These two “extreme” cases analysed (considering or neglecting particle Brownian motion) suggest that the particles experience similar transport phenomena inside the reactor at both the scales investigated, justifying the similar performance of the small and scaled-up systems.

## 4. Conclusions

Iron oxide nanoparticles were synthesized via oxidative hydrolysis of ${\mathrm{Fe}}^{2+}$ in the presence of a mild oxidant. This synthesis was carried out in four reactor systems, differing in terms of flow configuration (single- or two-phase flow) and presence of dissolved gas in the reaction solution or the segmenting fluid. The reactor systems were assembled using two distinct units, namely a tube-in-tube gas–liquid contactor, and a PTFE coil heated at the reaction temperature. These two modular units can be combined, allowing one to work either in single-phase or liquid–liquid segmented flow, as well as in the presence of gas. The use of a flow reactor allowed simple and safe access to a window of operating conditions hardly accessible in batch, such as temperatures above solvent boiling point, through the straightforward pressurization of the system. This significantly reduced the reaction time compared to equivalent batch syntheses. The use of gas–liquid tube-in-tube contactors holds great promise in accessing operating parameters windows not usually accessible in batch, as well as representing a potentially scalable technology. This has been demonstrated already for the synthesis of small molecules in organic synthesis, but represents an option still poorly explored for the flow synthesis of nanomaterials. Particle morphology and selectivity towards the desired magnetite/maghemite phases worsened when carrying out the synthesis in a liquid–liquid segmented flow reactor. Among the possible phenomena taking place in the multiphase system, interfacial adsorption at the aqueous-organic interphase might explain the observed deviations from the expected particle morphology and the high particle polydispersity, i.e., distinct particle populations within the same sample. On the other hand, this work also shows how the use of single-phase reactors represents a viable (or even better) option to carry out some nanomaterials synthesis instead of two-phase reactors: single-phase reactors (in certain cases) still allow fouling-free operation and at the same time do not require downstream separation. The presence of a reducing gas ($H_{2}$) improved the selectivity towards the magnetite/maghemite phases. The introduction of carbon monoxide in the reaction mixture resulted in smaller particles, presumably due to the adsorption of CO on the surface of the particles, hence offering a further degree of freedom to control the particle size in the synthesis. The work also reports the successful scale up by a factor of 5 of one of the developed reactor systems, namely the single-phase reactor where the solution was preventively bubbled with $N_{2}$, producing particles equivalent to those obtained in the smaller scale reactor. The similar behaviour of the two reactors (small- and large-scale) was justified through the analysis of the key dimensionless numbers describing the transport phenomena in both systems.

## Figures and Tables

**Table 1:** Summary of particle size, saturation magnetization, SAR and ILP of the particles produced using Reactor Systems 2, 3 and 4.

Reactor System	Particle Size (nm)	Saturation Magnetization (emug)	SAR (WgFe)	ILP (nHm2kg)
2 (two-phase flow with $H_{2}$-saturated heptane)	42±11	84	501 * 765 **	2.7 * 2.65 **
3 (single-phase flow with solutions preventively bubble with $N_{2}$)	34±4.8	79	220 * 395 **	1.25 * 1.3 **
4 (single-phase flow with CO-saturated base solution)	26.5±4.6	80	215 * 382 **	1.2 * 1.3 **

* 303 kHz, 24.6 kA/m; ** 759 kHz, 19.9 kA/m.

## Supplementary Materials

The following are available online at https://www.mdpi.com/1996-1944/13/4/1019/s1, Figure S1: (a) XRD patterns of the particles produced with the different reactor systems; magnification in the range (b) 45°–50° and (c) 60°–70°. The black vertical lines are the reference peaks for magnetite/maghemite (pdf ref. 03-065-3107) and the olive vertical lines are the reference peaks for feroxyhyte (pdf ref. 01-077-0247). The XRD pattern for Reactor System 1 shows features of both magnetite/maghemite and feroxyhyte; Reactor Systems 2, 3 and 4 match the reference peaks for magnetite/maghemite. Figure S2: Colour map of (a) hydrogen and (b) carbon monoxide concentration in the gas–liquid tube-in-tube contactor as used in Reactor System 2 and 4 respectively. Axial concentration profiles as mixed-cup averaged over the cross section of (c) hydrogen and (d) carbon monoxide in the gas–liquid tube-in-tube contactor in Reactor Systems 2 and 4 respectively. Figure S3: Magnetization curves obtained from VSM at a temperature of 300 K of the iron oxide nanoparticles produced using Reactor Systems 2, 3 and 4. Inset: magnification of the low field region of the curves to highlight their hysteresis loop. Figure S4: Temperature increase over time upon exposure of the colloidal solutions to an alternating magnetic field. Curves obtained from particles produced using (a) Reactor System 2, (b) Reactor System 3 and (c) Reactor System 4. The corresponding SAR and ILP values are also indicated. Figure S5: (a) XRD patterns of particles produced using the small- and large-scale version of Reactor System 3; the black vertical lines are the reference peaks for magnetite/maghemite (pdf ref. 03-065-3107); (b) heating curves of particles obtained from the large and small-scale reactors upon exposure to an alternate magnetic field; field frequency of 759 kHz and amplitude of 19.9 kA/m. Table S1: Parameters used in the model computations
