# Supplementary material for: A Modular Millifluidic Platform for the Synthesis of Iron Oxide Nanoparticles with Control over Dissolved Gas and Flow Configuration
Source: Materials (Basel). 2020 Feb 25;13(4):1019. doi: 10.3390/ma13041019 (PMC7079590; doi:10.3390/ma13041019)
Supplement: Supplementary file 1 [file materials-13-01019-s001.pdf]

Supplementary Information

A Modular Millifluidic Platform for the Synthesis of Iron Oxide Nanoparticles with Control over Dissolved Gas and Flow Configuration

Contents

SI 1 – XRD Spectra 2
SI 2 – Gas–liquid Tube-in-Tube Contactor Model 3
SI 3 – Magnetic Properties of Fe_3O_4 Nanoparticles 5
SI 4 – Heating Curves upon Alternating Magnetic Field Exposure 6
SI 5 – Comparison of Products obtained from Different Scale Reactors 9
References 10

SI 1 – XRD Spectra

XRD was used to determine the crystal structure of the particles produced with the various reactor systems described in the main text. All samples show a prevalence of magnetite/maghemite (Figure S1a). The XRD pattern of the particles produced with Reactor System 1 also shows features of feroxyhyte (Figure S1b,c).

(a)

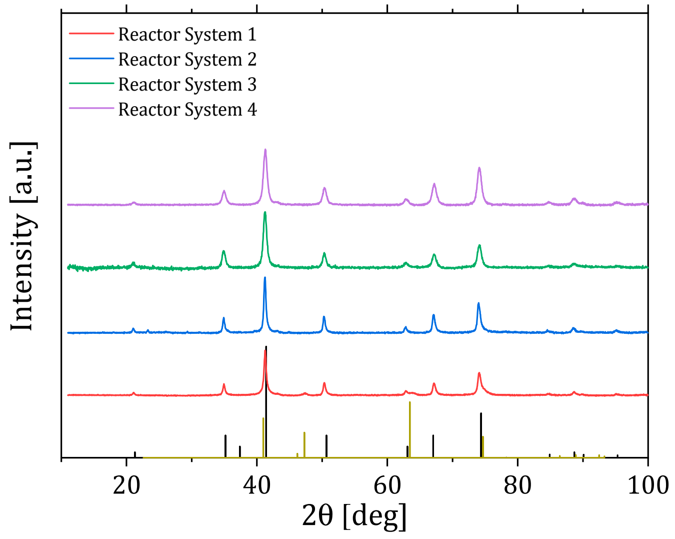

(b)

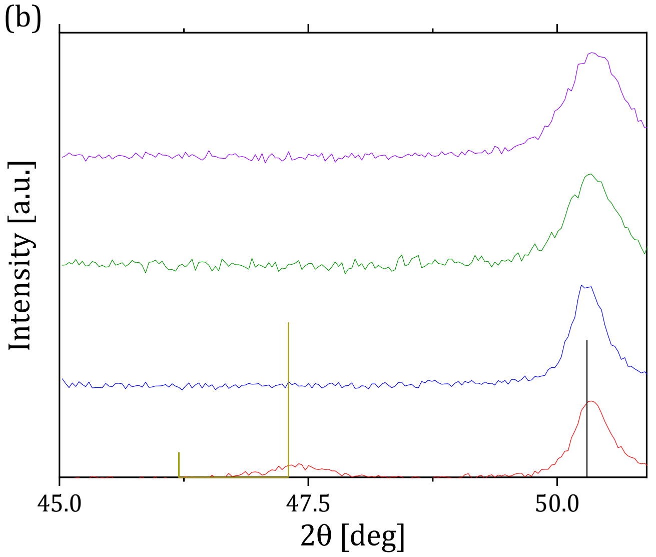

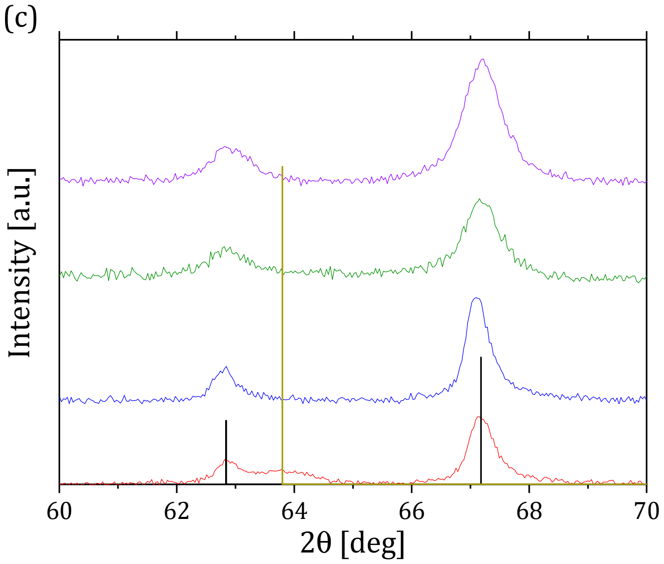

Figure S1. (**a**) XRD patterns of the particles produced with the different reactor systems; magnification in the range (**b**) 45°−50° and (**c**) 60°−70°. The black vertical lines are the reference peaks for magnetite/maghemite (pdf ref. 03-065-3107) and the olive vertical lines are the reference peaks for feroxyhyte (pdf ref. 01-077-0247). The XRD pattern for Reactor System 1 shows features of both magnetite/maghemite and feroxyhyte; Reactor Systems 2, 3 and 4 match the reference peaks for magnetite/maghemite.

SI 2 – Gas–liquid Tube-in-Tube Contactor Model

The model applied to design the tube-in-tube gas–liquid contactor was adapted from previous work [1,2], and the simulations were performed using COMSOL Multiphysics software (Version 5.2a). The following assumptions were made:

- steady-state mass transfer in an axisymmetric geometry at isothermal conditions;
- laminar flow (parabolic velocity profile with invariant axial position) in the inner tube;
- Henry's law applied to the membrane–liquid interface;
- ideal gas in gas phase;
- negligible liquid pervaporation through the membrane to the gas phase.

Table S1 summarizes the parameters used in the simulations. For Reactor System 2, the flow rate was set equal to $1\ \mathrm{mL/min}$, while for Reactor System 4, the flow rate was set equal to $0.5\ \mathrm{mL/min}$..

Table S1. Parameters used in the model computations.

Parameter	Value
Inner tube radius, R_{inner}	0.4 mm
Membrane thickness, δ	0.1 mm
Tube length	2 m
Diffusivity of gases in membrane*	$0.63 \times 10^{-9}\ \mathrm{m^2s^{-1}}$
Pressure	3 bar
Diffusivity of $\mathrm{H_2}$ in heptane [3]	$9.5 \times 10^{-9}\ \mathrm{m^2s^{-1}}$
Diffusivity of CO in water [4]	$2 \times 10^{-9}\ \mathrm{m^2s^{-1}}$
Henry constant of $\mathrm{H_2}$ in heptane [5]**	1.54×10^{3} bar
Henry constant of CO in water [6]	5.8×10^{4} bar

* As the membrane permeabilities for CO and H_2 are not available, nitrogen permeability was used for the estimation [2]; ** value obtained by interpolating between the constants of $\mathrm{H_2}$ in n-hexane and n-octane.

Figure S2 shows the concentration profile of the hydrogen in heptane and carbon monoxide in water inside the inner tube where the reaction mixture was flowing (Figure S2a,b), as well as along the reactor axis (Figure S2 c,d) obtained from the simulations.

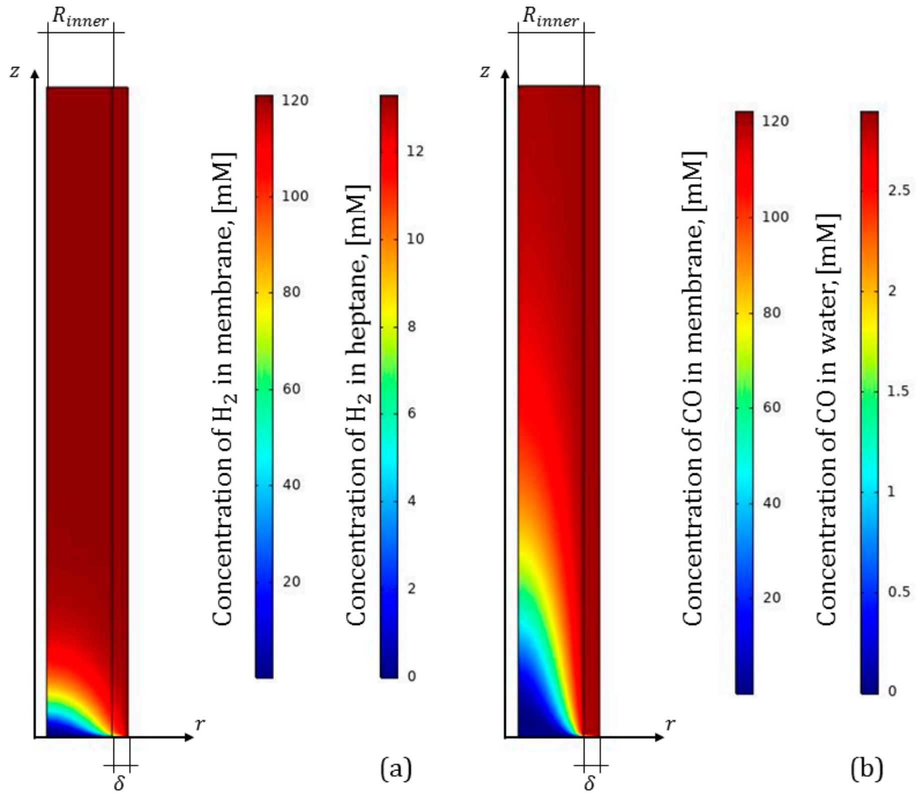

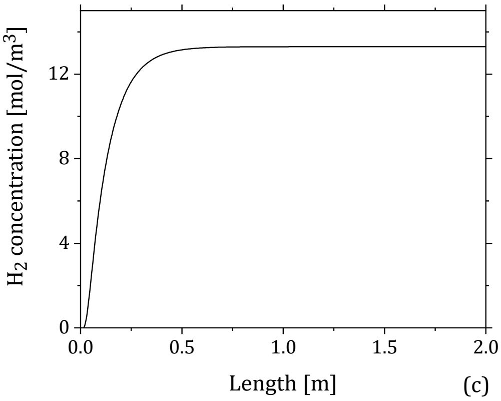

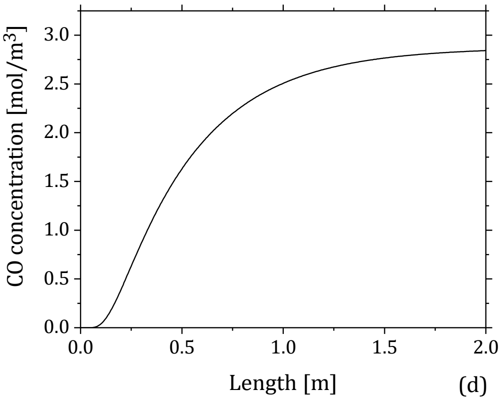

Figure S2. Colour map of (**a**) hydrogen and (**b**) carbon monoxide concentration in the gas–liquid tube-in-tube contactor as used in Reactor System 2 and 4 respectively. Axial concentration profiles as mixed-cup averaged over the cross section of (**c**) hydrogen and (**d**) carbon monoxide in the gas–liquid tube-in-tube contactor in Reactor Systems 2 and 4 respectively.

SI 3 – Magnetic Properties of Fe_3O_4 Nanoparticles

Vibrating sample magnetometry (VSM) was employed to determine the magnetic properties of the particles produced from Reactor Systems 2, 3 and 4 at room temperature up to 2.5 T. All the samples exhibited a saturation magnetization of ~80 emu/g, close to that of bulk magnetite (92 emu/g), supporting the phase purity of the particles produced. The particles exhibited a ferromagnetic behaviour, evidenced by a hysteresis loop (inset in Figure S3), with a coercivity increasing from ~2 mT (Reactor System 4, average particle size 26.5 nm) to ~4 mT (Reactor System 3, average particle size 34 nm) up to ~10 mT (Reactor System 2, average particle size 42 nm). These values are in line with those reported by Marciello et al. for similar particles [7].

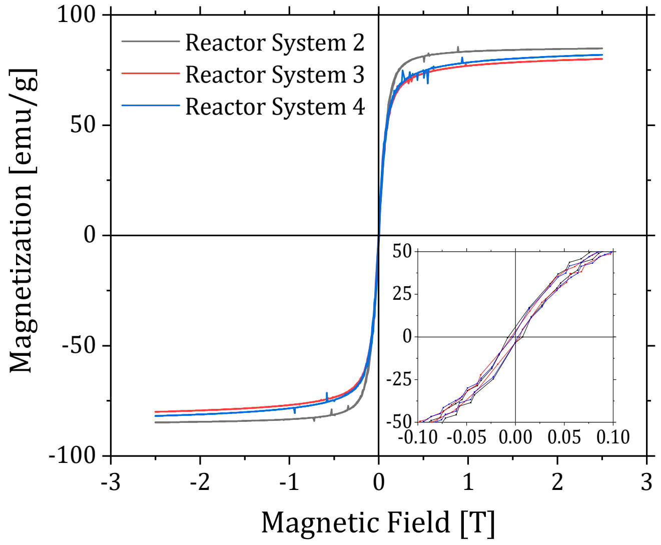

Figure S3. Magnetization curves obtained from VSM at a temperature of 300 K of the iron oxide nanoparticles produced using Reactor Systems 2, 3 and 4. Inset: magnification of the low field region of the curves to highlight their hysteresis loop.

SI 4 – Heating Curves upon Alternating Magnetic Field Exposure

Colloidal solutions of particles obtained from Reactor Systems 2, 3 and 4 were exposed to an alternate magnetic field in two different configurations (Frequency = 303 kHz, Field = 24.6 kA/m and Frequency = 759 kHz, Field = 19.9 kA/m) and the temperature of the colloidal solution was measured with the aid of a fibre optic placed inside the liquid sample. The results are reported in Figure S4.

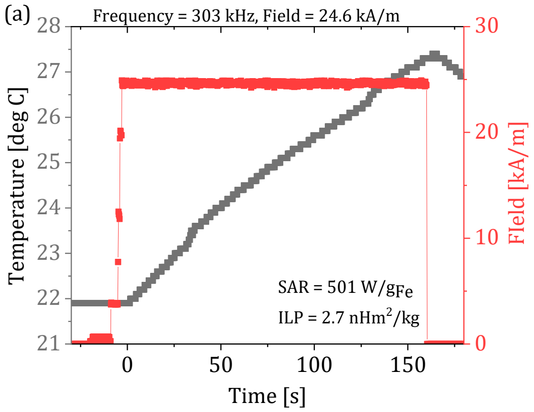
(a)
Frequency = 303 kHz, Field = 24.6 kA/m
Temperature [deg C]
Field [kA/m]
Time [s]
SAR = 501 W/gFe
ILP = 2.7 nHm2/kg

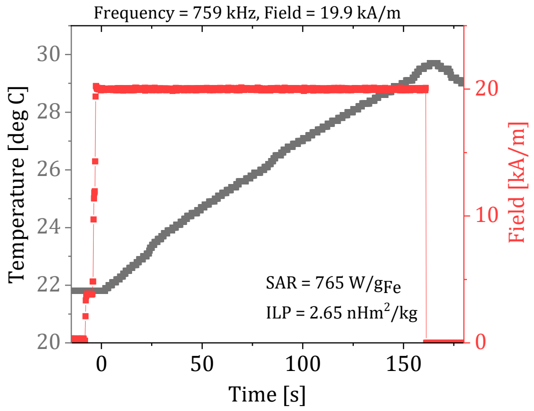
Frequency = 759 kHz, Field = 19.9 kA/m
Temperature [deg C]
Field [kA/m]
Time [s]
SAR = 765 W/gFe
ILP = 2.65 nHm2/kg

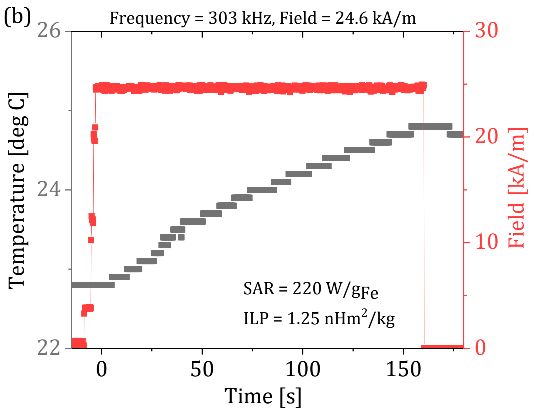
(b)
Frequency = 303 kHz, Field = 24.6 kA/m
Temperature [deg C]
Field [kA/m]
SAR = 220 W/g$_{Fe}$
ILP = 1.25 nHm^2/kg
Time [s]
22
24
26
0
10
20
30
0
50
100
150

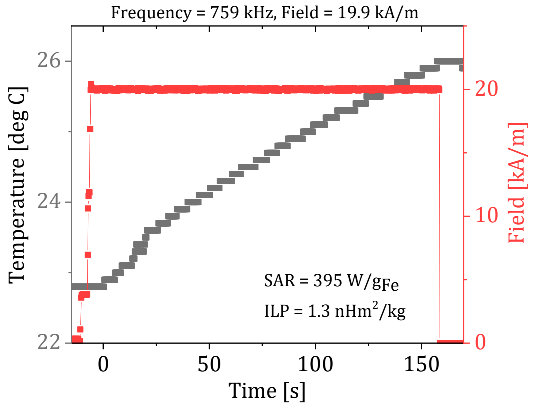
Frequency = 759 kHz, Field = 19.9 kA/m
Temperature [deg C]
Field [kA/m]
SAR = 395 W/g$_{Fe}$
ILP = 1.3 nHm^2/kg
Time [s]
22
24
26
0
10
20
0
50
100
150

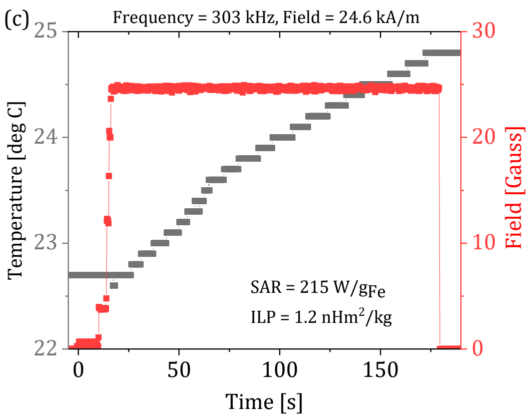

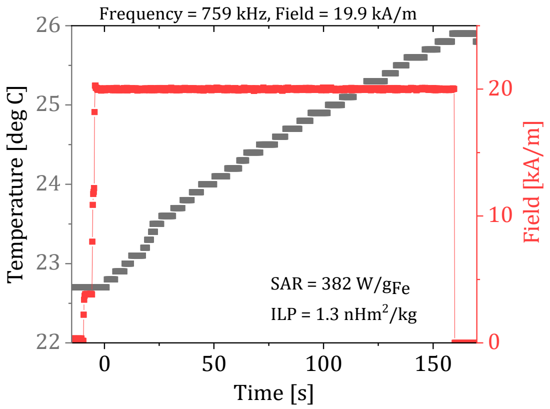

Figure S4. Temperature increase over time upon exposure of the colloidal solutions to an alternating magnetic field. Curves obtained from particles produced using (**a**) Reactor System 2, (**b**) Reactor System 3 and (**c**) Reactor System 4. The corresponding SAR and ILP values are also indicated.

SI 5 – Comparison of Products obtained from Different Scale Reactors

The particles produced with the scaled-up version of Reactor System 3 were analysed with XRD to determine the crystal structure of the product. No changes compared to the small-scale Reactor System were observed (Figure S5a). The particles were tested in solution as potential heaters upon exposure to an alternating magnetic field (Frequency = 759 kHz, Field = 19.9 kA/m), leading to equivalent results as from those produced using the small-scale Reactor System (Figure S5b).

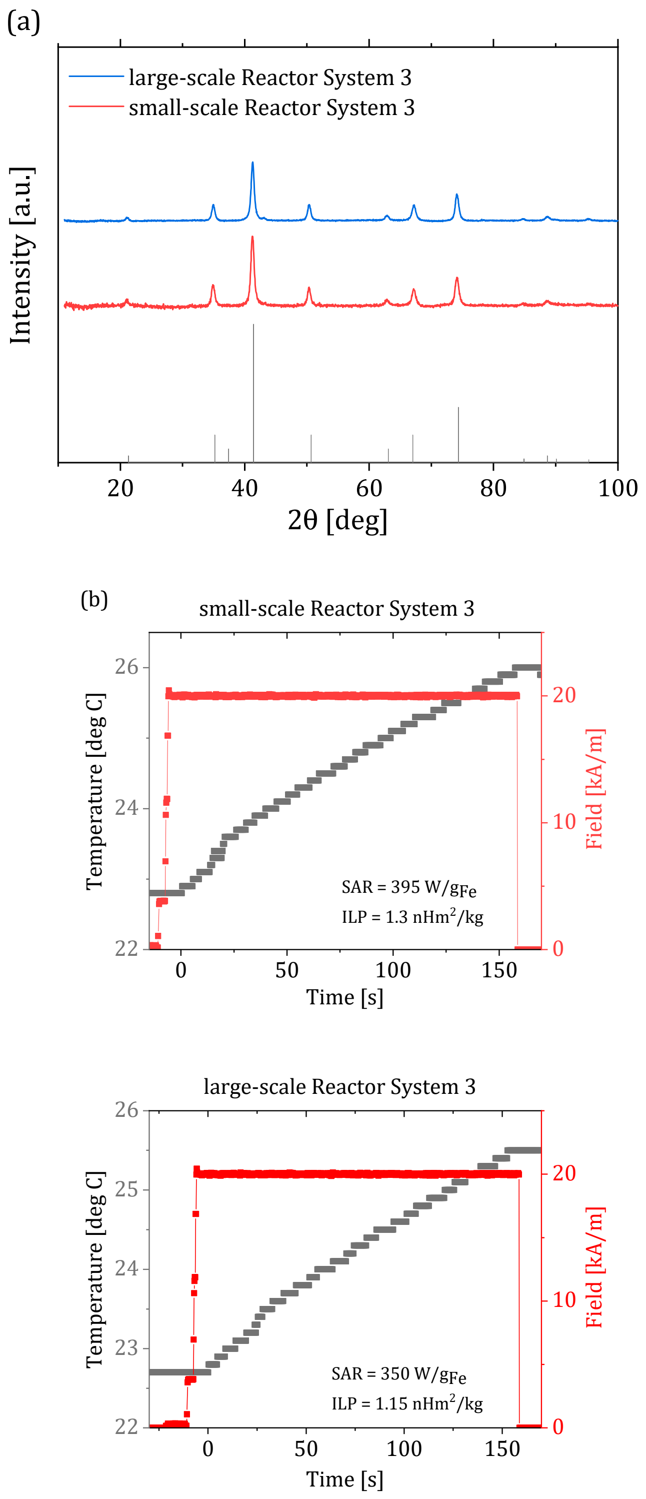

Figure S5. (**a**) XRD patterns of particles produced using the small- and large-scale version of Reactor System 3; the black vertical lines are the reference peaks for magnetite/maghemite (pdf ref. 03-065-3107); (**b**) heating curves of particles obtained from the large and small scale reactors upon exposure to an alternate magnetic field; field frequency of 759 kHz and amplitude of 19.9 kA/m.

References

1. Yang, L.; Jensen, K.F. Mass transport and reactions in the tube-in-tube reactor. *Org. Process Res. Dev.* **2013**, *17*, 927–933.

2. Huang, H.; Hwang, G.B.; Wu, G.; Karu, K.; Du Toit, H.; Wu, H.; Callison, J.; Parkin, I.P.; Gavriilidis, A. Rapid synthesis of [Au25(Cys)18] nanoclusters via carbon monoxide in microfluidic liquid-liquid segmented flow system and their antimicrobial performance. *Chem. Eng. J.* **2019** (in press).
3. Guo, Y.; Zhu, L.K.; Zhang, Y.P.; Liu, J.; Guo, J.S. Experimental study of mass diffusion coefficients of hydrogen in dimethyl phosphate and n-heptane. *IOP Conf. Ser. Earth Environ. Sci.* **2017**, *93*, 12057–12064.
4. Cussler, E.L. *Difussion - Mass Transfer in Fluid Systems*; 3rd ed.; Cambridge University Press: Cambridge, UK, 2009.
5. Brunner, E. Solubility of hydrogen in 10 organic solvents at 298.15, 323.15, and 373.15 K. *J. Chem. Eng. Data* **1985**, *30*, 269–273.
6. Sander, R. Compilation of Henry's law constants (version 4.0) for water as solvent. *Atmos. Chem. Phys.* **2015**, *15*, 4399–4981.
7. Marciello, M.; Connord, V.; Veintemillas-Verdaguer, S.; Vergés, M.A.; Carrey, J.; Respaud, M.; Serna, C.J.; Morales, M.P. Large scale production of biocompatible magnetite nanocrystals with high saturation magnetization values through green aqueous synthesis. *J. Mater. Chem. B* **2013**, *1*, 5995–6004.

© 2020 by the authors. Submitted for possible open access publication under the terms and conditions of the Creative Commons Attribution (CC BY) license (http://creativecommons.org/licenses/by/4.0/).
